# Phosphoinositide-signaling is one component of a robust plant defense response

**DOI:** 10.3389/fpls.2014.00267

**Published:** 2014-06-11

**Authors:** Chiu-Yueh Hung, Peter Aspesi Jr, Melissa R. Hunter, Aaron W. Lomax, Imara Y. Perera

**Affiliations:** Department of Plant and Microbial Biology, North Carolina State UniversityRaleigh, NC, USA

**Keywords:** Arabidopsis, Ca^2+^, salicylic acid, phosphoinositides, InsP_3_, plant defense signaling, SAR

## Abstract

The phosphoinositide pathway and inositol-1,4,5-triphosphate (InsP_3_) have been implicated in plant responses to many abiotic stresses; however, their role in response to biotic stress is not well characterized. In the current study, we show that both basal defense and systemic acquired resistance responses are affected in transgenic plants constitutively expressing the human type I inositol polyphosphate 5-phosphatase (InsP 5-ptase) which have greatly reduced InsP_3_ levels. Flagellin induced Ca^2+^-release as well as the expressions of some flg22 responsive genes were attenuated in the InsP 5-ptase plants. Furthermore, the InsP 5-ptase plants were more susceptible to virulent and avirulent strains of *Pseudomonas syringae* pv. *tomato* (*Pst*) *DC3000*. The InsP 5-ptase plants had lower basal salicylic acid (SA) levels and the induction of SAR in systemic leaves was reduced and delayed. Reciprocal exudate experiments showed that although the InsP 5-ptase plants produced equally effective molecules that could trigger *PR-1* gene expression in wild type plants, exudates collected from either wild type or InsP 5-ptase plants triggered less *PR-1* gene expression in InsP 5-ptase plants. Additionally, expression profiles indicated that several defense genes including *PR-1*, *PR-2*, *PR-5*, and *AIG1* were basally down regulated in the InsP 5-ptase plants compared with wild type. Upon pathogen attack, expression of these genes was either not induced or showed delayed induction in systemic leaves. Our study shows that phosphoinositide signaling is one component of the plant defense network and is involved in both basal and systemic responses. The dampening of InsP_3_-mediated signaling affects Ca^2+^ release, modulates defense gene expression and compromises plant defense responses.

## Introduction

Plants have developed a multilayered strategy to effectively control and combat pathogen invasion (Jones and Dangl, 2006). The first line of defense is triggered by the recognition of microbial- or pathogen-associated molecular patterns (MAMP or PAMP) by the membrane associated pattern recognition receptors (PRRs) (Nürnberger et al., 2004; Delledonne, 2005). Pathogen triggered immunity (PTI) also known as basal resistance (Jones and Dangl, 2006), is non-specific and against a range of virulent pathogens. A series of rapid responses are initiated within seconds to minutes of encountering MAMPs including ionic fluxes, production of reactive oxygen species (ROS) and activation of kinase cascades followed shortly by transcriptional reprogramming (reviewed in Postel and Kemmerling, 2009; Gimenez-Ibanez and Rathjen, 2010; Tsuda and Katagiri, 2010).

The second line of defense known as effector-triggered immunity (ETI) involves recognition of specific pathogen avirulent proteins by their counterpart plant disease resistance (R) proteins located within the cell (reviewed in Jones and Dangl, 2006). The local infected sites undergo programmed cell death to halt pathogen growth as part of the hypersensitive response (HR). HR also involves rapid ion fluxes and production of ROS (reviewed in Torres et al., 2006) as well as induction of pathogenesis-related (PR) proteins. In addition to the local response, distal parts of the plant develop immunity against a broad range of pathogens, known as systemic acquired resistance (SAR). A hallmark of SAR is an increase in salicylic acid (SA). SA accumulates in both distal and infected leaves in response to pathogen attack and treatment with SA or SA analogs can induce the *PR* genes such as *PR-1*, *PR-2*, and *PR-5* (reviewed in Durrant and Dong, 2004; Fu and Dong, 2013). More recently several other metabolites have been implicated as mobile signals in the development of SAR (Park et al., 2007; reviewed in Kachroo and Kachroo, 2009; Gao et al., 2014).

A transient increase in cytosolic Ca^2+^ ([Ca^2+^]_*in*_) is one of the early events associated with both PTI and ETI. The initial increase in Ca^2+^ upon MAMP recognition is accepted to be via an influx through plasma membrane localized Ca^2+^ channels, such as cyclic nucleotide gated channels (CNGC) and/or glutamate receptor (GluRs) channels. In addition, the involvement of intracellular Ca^2+^ stores in the propagation of the Ca^2+^ signal is very likely (reviewed in Ma and Berkowitz, 2007; McAinsh and Pittman, 2009; Manzoor et al., 2012). Several downstream defense related events are regulated by Ca^2+^ including activation of mitogen-activated protein kinase (MAPK) and calcium-dependent protein kinase (CDPK) cascades and modulation of Ca^2+^ binding proteins (reviewed in Ma and Berkowitz, 2011; Wurzinger et al., 2011; Boudsocq and Sheen, 2013; Romeis and Herde, 2014). The MAMP-triggered oxidative burst also appears to dependent on the initial cytosolic Ca^2+^ elevation. The NADP oxidase RbohD is directly and indirectly regulated by Ca^2+^ (Sagi and Fluhr, 2006; Dubiella et al., 2013). In response to elicitation with flg22, early membrane depolarization events are unaffected in the *rbohD* mutant (Jeworutzki et al., 2010) and the cytosolic Ca^2+^ elevation is similar to wild type plants (Ranf et al., 2011). A rise in cytosolic Ca^2+^ is also important for ETI responses and may precede the oxidative burst in response to avirulent pathogens (Grant et al., 2000). Furthermore, several Ca^2+^ or calmodulin (CaM) binding transcription factors TGA, CPB60g and CAMTA3, (reviewed in Reddy et al., 2011) act as both positive and negative regulators of SA accumulation and SA-regulated *PR* gene expression.

Many lipids and lipid related molecules are thought to play roles in plant defense signaling and responses to both biotrophic and necrotrophic pathogens (reviewed in Shah, 2005; Walley et al., 2013). However, there is less information on the potential involvement of the phosphoinositide signaling pathway in plant defense. The membrane-associated phospholipids along with the soluble inositol phosphates (collectively known as phosphoinositides) are present in all eukaryotic cells and are implicated in plant responses to many environmental stimuli (see reviews in Stevenson et al., 2000; Meijer and Munnik, 2003; Krinke et al., 2007a; Im et al., 2010, 2011). In the canonical pathway, phosphatidylinositol is sequentially phosphorylated to phosphatidylinositol 4-phosphate (PtdIns4P) and phosphatidylinositol 4,5-bisphosphate (PtdInsP_2_) by the enzymes phosphatidylinositol 4-kinase (PI4K) and phosphatidylinositol 4-phosphate 5-kinase (PIP5K), respectively. Activation of phospholipase C (PLC) in response to a stimulus or stress leads to the hydrolysis of PtdInsP_2_ to generate the soluble second messenger inositol 1,4,5-trisphosphate (InsP_3_) and diacylglycerol (DAG). While many aspects of the phosphoinositide pathway are conserved in plants there are several differences between plants and animals (reviewed in Munnik and Nielsen, 2011; Delage et al., 2013; Pokotylo et al., 2014). In plants, DAG is converted to phosphatidic acid (PtdOH), while InsP_3_ maybe further phosphorylated to form inositol hexakisphosphate (InsP_6_). Both InsP_3_ and InsP_6_ are thought to release Ca^2+^ from intracellular stores (reviewed in Krinke et al., 2007a; Im et al., 2010; Gillaspy, 2011). Additionally, InsP_5_ and InsP_6_ are implicated in hormone signaling via their interaction with the jasmonic acid receptor COI (Sheard et al., 2010; Mosblech et al., 2011) and the auxin receptor TIR1 (Tan et al., 2007), respectively. PtdOH may also be generated by hydrolysis of structural phospholipids such as phosphatidylcholine (PC) and phosphatidylethanolamine (PE) via the phospholipase D (PLD) enzymes. Recent reports implicate PtdOH as an important signaling molecule in plants in both abiotic and biotic stresses (reviewed in Testerink and Munnik, 2011). Using a transgenic inducible system to express type III effectors (AvrRpm1 or AvrRpt2) in planta, a biphasic accumulation of PtdOH was reported involving first PLC and then PLD activation (Andersson et al., 2006). Furthermore, activation of both PI4K and PLD has been shown to be an early response of suspension cells to treatment with SA (Krinke et al., 2007b, 2009). PtdOH-binding targets include the NAD oxidase RbohD and MPK6 protein kinase and PtdOH is implicated in ROS and HR responses (reviewed in Canonne et al., 2011; Testerink and Munnik, 2011). Specific PLC isoforms may also regulate defense responses (Vossen et al., 2010; Canonne et al., 2011). Arabidopsis mutants which have constitutively low InsP_6_ levels exhibited an increased susceptibility to microbial infections (Murphy et al., 2008). Collectively these results support the involvement of the phosphoinositides in plant defense; however there are many missing links in our understanding.

In this study, our goal was to further investigate the function of phosphoinositide-mediated signaling in the plant defense network. In previous work, we generated transgenic Arabidopsis plants expressing the human type I inositol polyphosphate 5-phosphatase (InsP 5-ptase), which specifically hydrolyzes soluble inositol phosphates and terminates InsP_3_-mediated signals. These InsP 5-ptase transgenic plants have normal growth and morphology under optimal growth conditions. However, their basal InsP_3_ levels are greatly reduced, to only ~5% of wild type level (Perera et al., 2006). The InsP 5-ptase plants exhibit delayed and reduced responses to gravity and impaired Ca^2+^ signaling in response to salt and cold stimuli (Perera et al., 2006, 2008). The InsP 5-ptase plants were also shown to be less resistant to wounding and herbivory (Mosblech et al., 2008). Interestingly, a global comparison of basal transcript profiles between wild type and InsP 5-ptase plants revealed that 16 out of 62 basally downregulated genes in transgenic plants were defense related (Perera et al., 2008), including *PR-1*, *PR-2*, *PR-5*, and *AIG1*(*avrRpt2- induced gene1*). In the current study, we demonstrate that InsP 5-ptase plants show impaired Ca^2+^ elevation in response to flg22, decreased expression of several defense related genes and delayed and reduced SAR. Our results support the role of InsP_3_ as a mediator of the intracellular Ca^2+^ cascade and highlight the involvement of phosphoinositide-mediated signaling in the development of a robust defense response in plants.

## Materials and methods

### Growth conditions for plants and bacteria

Transgenic Arabidopsis plants expressing the human type I inositol polyphosphate 5-phosphatase generated via Agrobacterium mediated transformation were generated previously (Perera et al., 2006). Plants were grown in a growth chamber under 8 h light/16 h dark, light intensity of ~150 μmol m^−2^ s^−1^, 35–50% humidity and temperature of 21°C. All experiments were carried out using 6–8 week old healthy well watered plants with fully expanded leaves (prior to bolting). For most experiments, wild type (Wt) and two independent transgenic lines (T6 and T8), were tested along with the empty vector control (C2). Bacterial strains *Pst* DC3000 and isogenic lines carrying *avrRpt2* or *avrRpm1*genes were cultured at room temperature in King's medium B (40 g L^−1^ peptone, 2% (v/v) glycerol, 1.5 g L^−1^ K_2_HPO_4_ and 1.5 g L^−1^ MgSO_4_, pH 7) containing 50 μg ml^−1^ kanamycin, and 100 μg ml^−1^ rifampacin. The two non-pathogenic strains, *Pst*DC3000 with mutated effector protein *hrcC*^−^ and *P. syringae* pv. *Phaseolicola* 6, were also cultured at room temperature on King's medium B plates containing 100 ug ml^−^ rifampacin, and an additional 34 μg ml^−1^ chloramphenicol for growing *Pst* DC3000 *hrcC*^−^.

### Pathogen inoculation

For preparing bacteria used in pathogen inoculation experiments, cells from an overnight culture were first resuspended in 10 mM MgCl_2_, and the concentration of bacteria was adjusted based on the absorbance reading at OD_600_. The actual titer was also determined by counting serial dilutions on a selection plate after 48 h at room temperature. To examine the growth of bacteria in planta, leaves were inoculated either by hand using a 1 ml needle-less syringe or by spraying with bacteria solution containing an additional 0.02% (v/v) silwet L-77 (Lehle Seeds, Round Rock, TX). The bacterial titer was determined from 4 leaf discs (total area is 1 cm^2^) collected from 4 separate leaves, (one leaf per plant). Pooled leaf discs were ground in 10 mM MgCl_2_ in a final volume of 1 ml. The colony-forming units (cfu) were obtained by spotting 2 μl of a serial 10-fold titrated bacteria onto King's medium B plates containing appropriate antibiotics and additional 50 μg ml^−1^ cyclohexamine for preventing fungus contamination. In each experiment, a total of 4 plants per line were used. For statistical analysis either Student's *t*-test or analysis of variance (ANOVA) were performed.

### Flagellin treatment

The elicitor flg22 was synthesized by EZBiolab custom peptide service (Westfield, IN) based on the peptide sequence reported in Felix et al. (1999). For root growth inhibition experiments, 0.1 μM of flg22 was used which caused the half-maximal growth inhibition in Arabidopsis seedlings reported in Gomez-Gomez et al. (1999). For induction of ROS production in leaf discs, the procedure was adapted from Gomez-Gomez et al. (1999) with a flg22 concentration of 1 μM. For induction of Ca^2+^ release and qRT-PCR analysis of flg22 responsive gene expression, 10 μM flg22 was used according to Navarro et al. (2004) and Zipfel et al. (2004).

### SAR assay

To induce SAR, two fully expanded leaves of wild type and InsP 5-ptase plants were hand inoculated with avirulent strain *Pst* DC3000+*avrRpt2* (OD_600_ = 0.001; 7.5 × 10^5^ cfu/ml). The un-induced plants were mock inoculated with 10 mM MgCl_2_, (two leaves per plant). Both induced and un-induced plants were then separated into 4 groups for challenging with the virulent strain *Pst*DC3000 (OD_600_ = 0.004; 2 × 10^6^ cfu/ml) at day 0, 1, 2, or 3 post inoculation by spraying the bacteria onto the whole plants. To examine the development of SAR, systemic leaves sprayed with *Pst*DC3000 were collected. Leaf extracts were prepared and the cfu was counted as described previously. The growth of virulent bacteria in induced and un-induced plants was compared for determining the level of SAR.

### Inositol (1,4,5) trisphosphate assay

For measuring the InsP_3_ level of inoculated leaves, 4–5 plants per experiment for each bacterial strain were grouped together and sprayed with bacterial solution prepared as before. The treated leaves were harvested and immediately frozen in liquid nitrogen. Frozen tissues (around 0.08 g) were ground into powder in liquid nitrogen and then incubated with 160 μl of 20% perchloric acid on ice for 15 min. Samples were centrifuged to remove the debris and the supernatant was transferred to a new tube followed by pH adjustment to 7.5 using 1.5 M KOH/3 mM HEPES. InsP_3_ assays were carried out using the TRK_1000_ InsP_3_ assay kit (Amersham-Pharmacia Biotech) as described previously (Perera et al., 2006).

### SA measurement and application

For analysis of total SA at the local inoculated leaves, plants were syringe-inoculated with *Pst*DC3000+*avrRpt2*, and inoculated leaves were harvested at day 2 post inoculation. To extract total SA (free SA + SA conjugates), the protocol described in Nandi et al. (2004) was used. In brief, leaves were first ground into powder in liquid nitrogen, and then ~0.2 g of ground tissue was extracted first with 90% methanol and again with 100% methanol. The combined extracts were subjected to evaporation under N_2_ gas. After evaporation, the residue was first resuspended in 5% trichloroacetic acid and then submitted to acid hydrolysis by HCl at 100°C for 30 min. SA was extracted with cyclohexane:ethylacetate:isopropanol (50:50:1). The organic phase was evaporated and resuspended in HPLC eluent. Chromatography was performed on a 3.9 × 300-mm C18 reverse-phase μBondapak column (Waters, Milford, MA, USA).

For exogenous SA application, SA (Sigma-Aldrich, St. Louis, MO) was first dissolved in ethanol, then diluted to 300 μM in water with additional 0.02% (v/v) silwet L-77 (Lehle Seeds, Round Rock, TX), and the pH was adjusted to 7 with 0.1 M KOH. For control solution, equal amount of ethanol without SA was diluted in the same way. Solutions were gently and evenly sprayed on plants. Treated plants were then kept in a growth chamber with plastic covers until harvest.

### Calcium-dependent aequorin bioluminescence

The wild type and two independent InsP 5-ptase plants (T6 and T8) expressing cytosolic aequorin (Knight et al., 1996) were created previously (Perera et al., 2008). For measuring Ca^2+^ changes after treatment with flg22, 5-day old seedlings were first incubated with 2 μM coelenterazine (Molecular Probe^®^, Eugene, OR) solution in the dark for 16 h. Seedlings were transferred into tubes containing water and placed in the luminometer and a baseline reading was obtained. After 1 min, seedlings were treated with 10 μM flg22 which was injected automatically to avoid any handling. The luminescence signals were recorded at 10 s intervals for 35 min. The Ca^2+^ concentration were calculated after measuring the discharged Ca^2+^ as described previously (Knight et al., 1996; Perera et al., 2008). For monitoring the Ca^2+^ increase after pathogen treatment, fully expanded leaves were first infiltrated with 2 μM coelenterazine (Molecular Probe^®^, Eugene, OR) solution and kept in the dark for 16 h, then the reconstituted leaves were inoculated with *Pst* DC3000+*avrRpm1* (OD_600_ = 0.5; 5 × 10^8^ cfu/ml) or 10 mM MgCl_2_ mock solution. Immediately, detached leaves were placed into a tube containing 100 μl water to prevent drying out. The luminescence signals were recorded every 5 s for 3 h. The timing of the peak luminescence were taken for calculation. Luminescence measurements were made using a Sirius luminometer (Berthold Detection Systems GmbH, Pforzheim, Germany).

### Chemiluminscent and *in situ* detection of ROS

For chemiluminescent detection of ROS, uniform leaf discs were first incubated overnight in sterile water in the dark. Two leaf discs were placed in a test tube containing 105 μL sterile water, with 1 μg horseradish peroxidase (HRP), 20 μM luminol, and 1μM flg22. Luminescence resulting from the reaction between hydrogen peroxide, HRP, and luminol was measured for 35 min in Sirius luminometer (Berthold Detection Systems GmbH, Pforzheim, Germany). For *in situ* detection of ROS, plants were either sprayed or hand-inoculated with *Pst*DC3000+*avrRpt2*. Infected leaves were carefully removed at 6 or 48 h, and vacuum-infiltrated with fresh 0.1% (w/v) of 3,3′-diaminobenzidine (DAB) (Sigma-Aldrich, St. Lious, MO) dissolved in 0.01 N HCl. Infiltrated leaves were kept in the dark for 3 h, then de-stained in a destaining solution of 95% EtOH:85% acetic acids: glycerol (3:1:1 in volume) and photographed.

### RNA isolation and qRT-PCR

Total RNA was isolated by RNeasy plant mini kits (Qiagen, Germantown, MD). For RT-PCR, the first strand cDNA was synthesized from total RNA using Ominiscript reverse transcriptase (Qiagen) and random primers, and PCR of specific genes was carried out using Hot Start Taq DNA polymerase (Qiagen). For qRT-PCR, the first strand cDNA was synthesized using StrataScript QPCR cDNA synthesis kits (Agilent Technologies Inc., Santa Clara, CA) and random primers, and the PCR reaction was performed using Full Velocity SYBR-Green QPCR Master mix (Agilent Technologies Inc.) on the Mx3000p thermocycler (Agilent Technologies Inc.). Genes that were analyzed from the Primary Library for Arabidopsis Pathogen-Inducible Genes (cat# PR0100, Sigma-Aldrich, St. Lious, MO) are listed in Supplementary Table 1. Primer sequences of other examined genes are listed in Supplementary Table 2. The fold change in relative gene expression was calculated based on the 2^−ΔΔCt^ method (Schmittgen and Livak, 2008) using wild type as the normalizer and *ACT2* or *PP2A* as the reference genes.

### Petiole exudate experiment

For collecting the petiole exudates, we followed the method described in Maldonado et al. (2002) and Chaturvedi et al. (2008). Leaves were hand inoculated with *Pst*DC3000+*avrRpt2* (OD_600_ = 0.01; 5.5 × 10^6^ cfu/ml) or mock inoculation with 10 mM MgCl_2_. Treated plants were placed in the growth chamber and covered with plastic lids. After 6–7 h, inoculated leaves were cut from their bases and immediately dipped in 50% ethanol. They were rinsed once briefly with 0.0005% Clorox bleach and then placed in 1 mM EDTA solution. A group of 10 leaves was placed in a collection tube containing 2.5 ml of 1mM EDTA and 50 μg ml^−1^ ampicillin, pH 8. They were kept in a covered tray with wet paper towels at the base of the tray to maintain the humidity in the growth chamber under continuous light. After 16 h, collected exudates were diluted 2 fold with water, and filter-sterilized before use.

## Results

### The response of InsP 5-ptase plants to flg22

**Figure 1:**
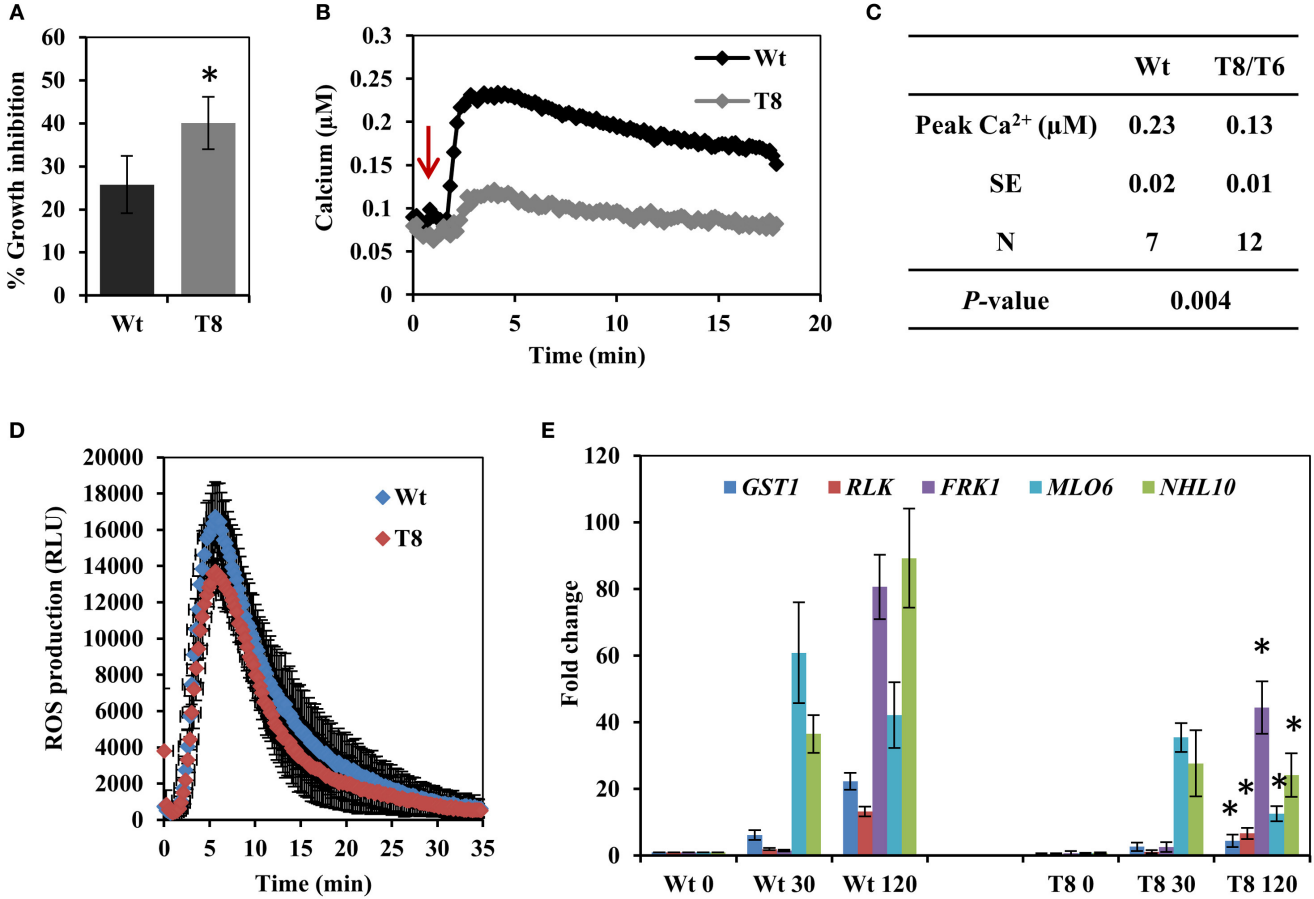
**The response of the InsP 5-ptase plants to flagellin. (A)** One week old wild type (Wt) and InsP 5-ptase (T8) seedlings were incubated in media containing either 0 or 0.1 μM flg22. Root length was measured after 5 days. Growth inhibition was calculated as the percentage of the non-treated control. Data is the average of four independent experiments (*n* = 10 seedlings per experiment) ± SE. **(B)** Five-day old seedlings expressing aequorin were reconstituted with coelenterazine overnight and placed in the luminometer. Seedlings were treated with 10 μM flg22 after 1 min (indicated by red arrow). Ca^2+^ concentrations were calculated after measuring the discharged Ca^2+^. A representative trace is shown. **(C)** The table lists the average peak Ca^2+^ concentration. (N = independent biological replicates). The average baseline concentrations were 0.097 and 0.098 for wild type and transgenic respectively. **(D)** ROS production was measured in leaf discs treated with 1 μM flg22. Luminescence was measured at 14 s intervals. Data plotted is the average of five biological replicates ± SD. **(E)** qRT-PCR analysis of flg22 responsive gene expression at 30 and 120 min post treatment. Data plotted is the average ± SE normalized to Wt control from three independent experiments. ^*^Indicates significant difference between Wt and T8 expression at the same time point (*P* < 0.05).

The bacterial elicitor flagellin is a well characterized MAMP used to investigate plant innate immunity responses. We first treated wild type and InsP 5-ptase seedlings with the active elicitor peptide flg22 and measured root growth. A greater inhibition of root growth was observed in the InsP 5-ptase seedlings compared to wild type (Figure 1A), indicating that the transgenic plants are more sensitive to flg22. We next monitored Ca^2+^ induction, ROS production and flg22-associated pathogen-responsive gene expression; which are all known to be triggered by flg22 treatment in Arabidopsis (Gomez-Gomez et al., 1999; Asai et al., 2002; Navarro et al., 2004; Zipfel et al., 2004; Boudsocq et al., 2010; Ma et al., 2012). Treatment with flg22 elicits a rapid rise in cytosolic Ca^2+^ which can be monitored using aequorin-mediated bioluminescence (Jeworutzki et al., 2010; Ranf et al., 2011). InsP 5-ptase seedlings expressing aequorin (Perera et al., 2008) were treated with flg22 and the intracellular Ca^2+^ changes were monitored. Upon treatment with flg22, a rapid rise in [Ca^2+^]_*cyt*_ with a peak at ~2 min was observed in both wild type and InsP 5-ptase plants; however, the induction level was greatly reduced in InsP 5-ptase plants (Figure 1B) with an average reduction in the Ca^2+^ signal of ~44% in the transgenic plants (Figure 1C). As shown by the root growth experiment (Figure 1A), the InsP 5-ptase plants are responsive to flg22; the increased root growth inhibition correlates with the attenuation of the Ca^2+^ signal. We also monitored the oxidative burst (by measuring H_2_O_2_ generation) after elicitation with flg22. An increase in ROS was detected at the first 1–2 min post treatment, which peaked ~5 min, and tapered off returning to the baseline within 25 min (Figure 1D), which was different from the sustained Ca^2+^ elevation (Figure 1B). Surprisingly, there was no significant difference in ROS generation between wild type and InsP 5-ptase plants. These results demonstrate that although the Ca^2+^ release after flg22 treatment was dampened, the oxidative burst was not affected.

Our data suggested that the InsP 5-ptase plants could be a tool to potentially distinguish between parallel downstream pathways of MAMP-triggered immunity. In order to further investigate the downstream responses, a group of genes known to be induced by flg22-FLS2 interaction (Asai et al., 2002; Qutob et al., 2006; Gust et al., 2007; Boudsocq et al., 2010) were selected and their expression levels were quantified by qRT-PCR. The five selected genes were *GST1*, *RLK*, *FRK1*, *NHL10*, and *MLO6*. The peak expression of *MLO6* was at 30 min after flg22 treatment, whereas the other four genes showed a peak expression at 120 min post flg22 treatment (Figure 1E). Both wild type and InsP 5-ptase plants, showed similar kinetics of induction; however, expression levels at 120 min were significantly reduced in the InsP 5-ptase plants compared to the wild type. The reduction in expression suggests that the InsP 5-ptase plants are unable to sustain or maintain maximal gene induction. Interestingly, *FRK1* and *NHL10* are both considered to be regulated via MAPK dependent pathways (Boudsocq et al., 2010) and these results suggest a role for phosphoinositide signaling interacting with the MAPK cascade.

### The response of InsP 5-ptase plants to virulent and avirulent strains of *P. syringae*

We next examined the interaction between the InsP 5-ptase transgenic plants and the plant pathogenic bacteria, *P. syringae* pv. *tomato* (*Pst*) DC3000 which is widely used to study plant disease resistance (Nishimura and Dangl, 2010). In Arabidopsis, *Pst*DC3000 bacteria multiply rapidly and disease symptoms can be observed ~2 day post-infection with characteristic tissue necrosis and chlorosis (Katagiri et al., 2002). Both virulent and avirulent strains of *P. syringae* were used to evaluate the susceptibility of InsP 5-ptase transgenic plants compared to wild type.

**Figure 2:**
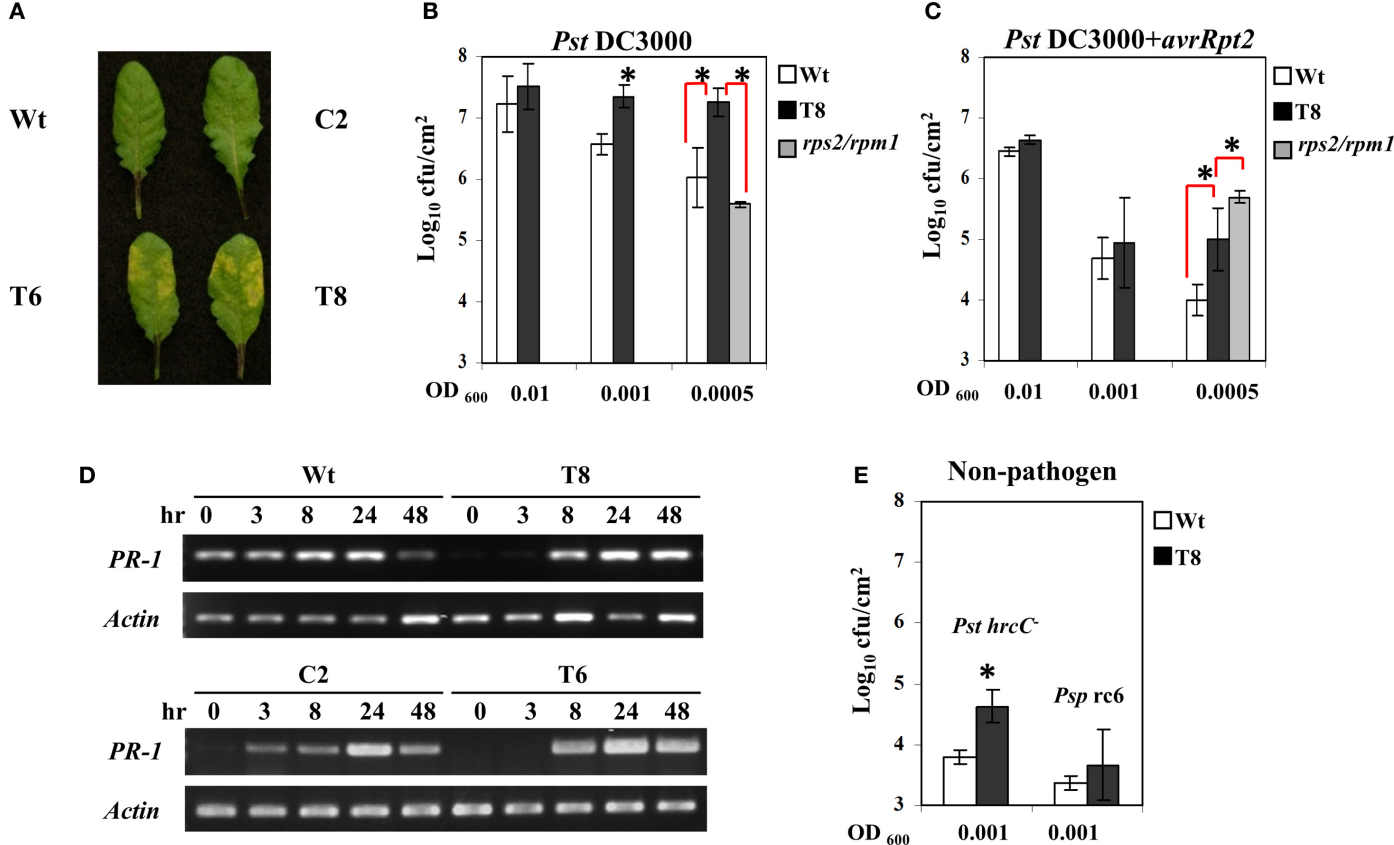
**The response of InsP 5-ptase plants to *Pseudomonas syringae***. Leaves of Arabidopsis plants inoculated with *Pst* DC3000 at OD_600_ = 0.001 (7 × 10^5^ cfu/ml) were photographed at day 2 after inoculation **(A)**. Bacterial growth measured from plants at day 2 after inoculation with *Pst* DC3000 **(B)** or *Pst*DC3000+*avrRpt2* **(C)**. Starting bacterial cultures are OD_600_ = 0.01 (8 × 10^6^ cfu/ml), 0.001 (7 × 10^5^ cfu/ml) or 0.0005 (3.5 × 10^5^ cfu/ml). **(D)** Infected leaves used for RT-PCR were inoculated with *Pst*DC3000+*avrRpt2* (OD_600_ = 0.001, 7 × 10^5^ cfu/ml). RT-PCR was carried out with gene-specific primers for *PR-1* and *Actin*. **(E)** For bacterial growth counts taken at day 4 after inoculation with the two non-pathogens, *Pst*DC3000+*hrcC*- (*Pst hrcC*-) and *P. syringae* pv. *Phaseolicola* race 6 (*Psp rc6*), the concentration of inoculants were OD_600_ = 0.001 (7 × 10^5^ cfu/ml). Plant lines used were Wt (white), T8 (black), and the *rps2/rpm1* double knock-out mutant (gray). ^*^P < 0.05.

Figure 2A shows that transgenic plants exhibited more severe symptoms after infection with the virulent strain of *P. syringae* (*Pst* DC3000). Moreover, when those infected leaves were harvested and bacterial concentration was quantified, the InsP 5-ptase transgenic plants harbored more bacteria at OD_600_ = 0.001 and 0.0005 compared to the wild type, indicating they are more susceptible to *Pst* DC3000 (Figure 2B). Similarly, the transgenic plants also showed a slight elevated bacterial growth of the avirulent strain *Pst*DC3000+*avrRpt2* at the low concentration of inoculants OD_600_ = 0.0005 (Figure 2C). Compared to the wild type the InsP 5-ptase plants are more susceptible to the avirulent strain; however the response to the avirulent strain was not as intense as the response to the virulent strain, suggesting that the gene-for-gene defense mechanism is still functional.

We also compared the disease response of InsP 5-ptase plants to that of *rps2/rpm1* double mutants (lacking both of the *Rps2* and *Rpm1 R* genes) which are equally susceptible to both virulent and avirulent strains of *Pst*DC3000 even at low concentrations of inoculant. We found that transgenic plants were less affected than the *rps2/rpm1* mutants when challenged with avirulent strains *Pst*DC3000+*avrRpt2*; and did not have the same high level of bacterial growth (Figure 2C). Therefore, the response of InsP 5-ptase plants to *Pst*DC3000+*avrRpt2* is not similar to *rps2/rpm1* double mutant which lacks the *R* gene mediated resistance. The enhanced susceptibility pattern observed in InsP 5-ptase plants is more similar to previously reported mutants with a defect in SA-mediated disease resistance, such as *nahG* and *eds16*/*sid2* mutants (Gaffney et al., 1993; Delaney et al., 1994; Dewdney et al., 2000; Wildermuth et al., 2001). These plants with a defect in SA-mediated resistance are more susceptible to avirulent bacterial strains, but not so severely as to virulent bacterial strains, indicating that the *R* genes in these mutant plants are not affected (Dewdney et al., 2000). It is possible that the enhanced susceptibility toward avirulent strains might be the result of dampened signal transduction leading to disease resistance. To test this hypothesis, we examined *PR-1* gene induction as a downstream response during the course of infection with *Pst*DC3000+*avrRpt2* at 3, 8, 24, and 48 h, and found that the two independent InsP 5-ptase lines T6 and T8 show a delayed induction of gene expression (the response was observed at 8 h instead of 3 h) compared to the wild type and vector control plants C2 (Figure 2D).

To further demonstrate that basal defense is compromised, we challenged InsP 5-ptase plants with a TTSS-defective *hrcC* mutant of *Pst*DC3000 (in which pathogen effector proteins are not delivered into host plants such that only a few bacteria multiply in wild type plants). We found a slight elevation in bacterial growth in InsP 5-ptase plants (Figure 2E), suggesting that the basal defense in InsP 5-ptase plants was weakened. However, we did not observe any bacterial growth or symptoms when challenged with the non-host pathogen strain *P. syringae* pv. *Phaseolicola* strain race 6 in both wild type and transgenic plants, even after 12 days (Figure 2E). These results suggest that the non-host resistance mechanism in transgenic plants is not affected.

Previous microarray data indicated that a group of disease resistance-related genes are basally downregulated under normal growth conditions in two independent InsP 5-ptase plants compared to wild type and vector control plants. These include *PR-1*, *PR-5*, and *AIG1*(*avrRpt2- induced gene1*), as well as genes encoding proteins involved in Ca^2+^ storage in the endoplasmic reticulum (ER) such as *CRT3* and *BiP3* (Perera et al., 2008). In this study we used qRT-PCR to analyze a set of selected genes. We determined that, in addition to previously identified defense-related genes, *PR-2*, two putative glutathione-S-transferase genes (*GST11* and *GST16*) and three protein kinases (*CRK7*, *CRK45*, and *RLP23*) were also basally downregulated in InsP 5-ptase plants (Table 1). Among the eleven genes tested, eight of them are also required for SAR according to the GO annotation in TAIR. Although *BiP3* has not been annotated as the other eight genes, other BiP isoforms have been shown to play a role in promoting plant immunity (Wang et al., 2005; Carvalho et al., 2014). The data suggest that reduced expression of these genes might contribute to the enhanced susceptibility of the transgenic plants to *P. syringae*.

**Table 1:** **Genes that were basally repressed in InsP 5-ptase plants**.

**Related function**	**TAIR ID**	**Gene^a^**	**Fold reduction^b^**
Defense genes	At2g14610	***PR-1***	33.3
	At3g57260	***PR-2***	8.3
	At1g75040	***PR-5***	5.0
	At1g33960	***AIG1***	4.8
Detoxification	At1g02920	*GST11*	4.0
	At2g02930	*GST16*	2.8
Protein folding	At1g08450	***CRT3***	3.1
	At1g09080	*BIP3*	10.0
Protein kinase	At4g11890	***ARCK1(CRK45)***	3.2
	At4g23150	***RLK7(CRK7)***	7.7
	At2g32680	***RLP23***	10.0

a The genes in bold are known to be involved in systemic acquired resistance based on Arabidopsis GO annotations.

b Data presented is the average fold change of three independent experiments (P < 0.05) for InsP 5-ptase transgenic line T8. Similar results were obtained with an independent line T6.

### InsP_3_ changes and calcium release during ETI

**Figure 3:**
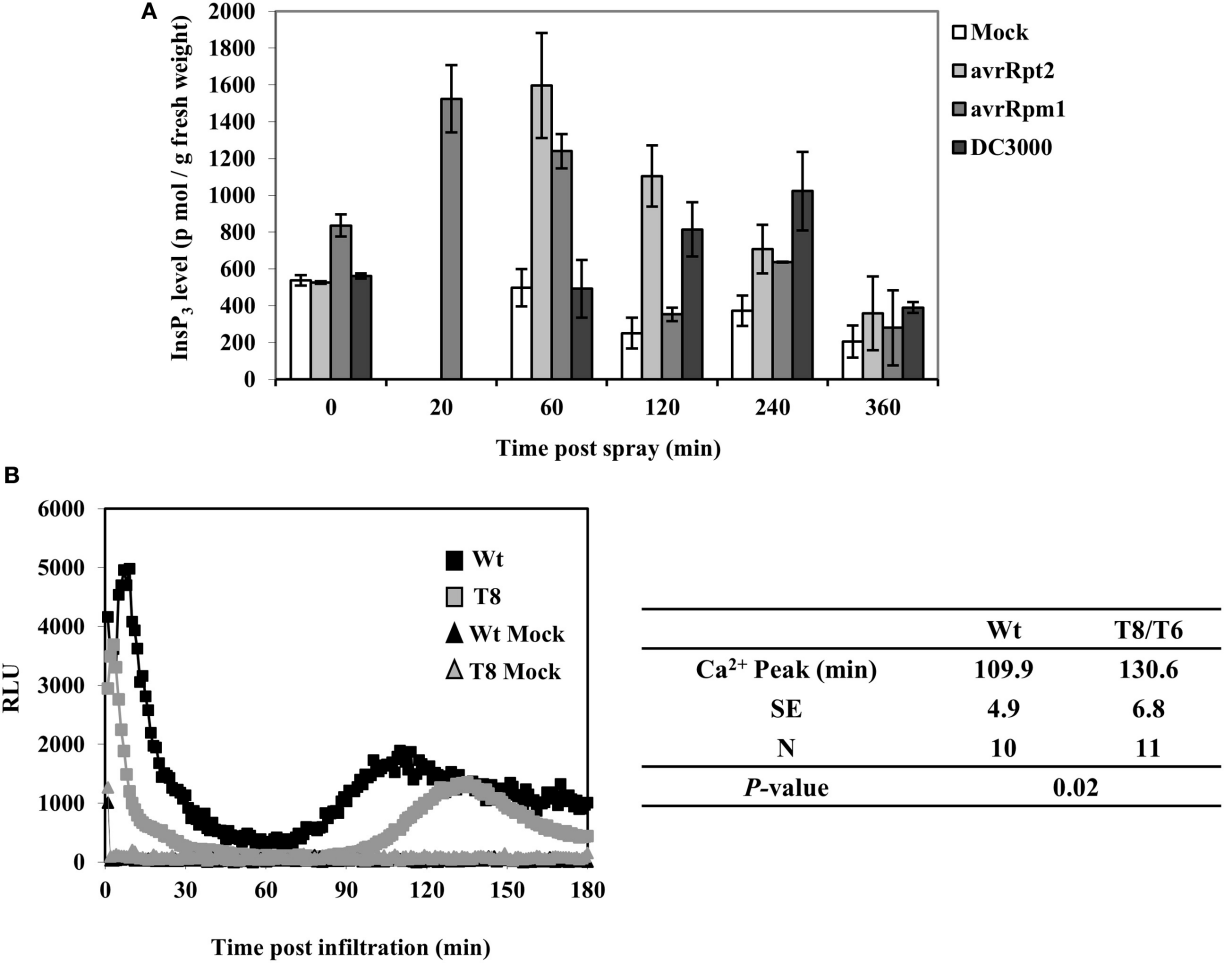
**InsP_3_ changes and Ca^2+^ release in response to avirulent *Pseudomonas syringae* pv *tomato* DC3000. (A)** Wild type Arabidopsis plants were sprayed with *Pst* DC3000 or *Pst* DC3000+*avrRpt2* or + *avrRpm1* at a concentration of OD_600_ = 0.004 (2 × 10^6^ cfu/ml). Mock spray was 10 mM MgCl_2_. Treated leaves were harvested at different time points and InsP_3_ was quantified. Data shown is the average of 5 independent experiments ± SE. **(B)** Wild type (Wt) or InsP 5-ptase seedlings carrying aequorin were reconstituted with coelenterazine and inoculation with *Pst* DC3000+*avrRpm1* (OD_600_ = 0.5, 5 × 10^8^ cfu/ml) or mock solution (10 mM MgCl_2_). A representative experiment is shown with luminescence counts taken in every 5 s. The table lists the average time of the second sustained Ca^2+^ peak (N = independent biological replicates).

In order to determine whether InsP_3_ is induced in wild type plants when infected with *P. syringae*, we examined InsP_3_ levels during a 6 h period of post spray with *P. syringae* in wild type Arabidopsis leaves. When spray-infected with a virulent strain *Pst* DC3000, InsP_3_ levels increased ~two-fold at 4 h post spray (Figure 3A). When infected with avirulent strains *Pst*DC3000+*avrRpm1* or *avrRpt2*, InsP_3_ levels increased 2–3 fold at a shorter time, 20 min and 1 h post spray, respectively. No change in InsP_3_ levels was observed with mock spray (Figure 3A). These results demonstrate that the induction of InsP_3_ is one of the signals generated during plant-pathogen interaction. The difference in the induction times of InsP_3_ between virulent and avirulent pathogens is in good agreement with the rapid induction of HR by avirulent strains compared to the slower development of disease symptoms by virulent strains (Katagiri et al., 2002).

In a previous study, a biphasic increase in cytosolic Ca^2+^ was measured by aequorin-mediated bioluminescence in Arabidopsis leaves infected with avirulent strains of *Pst*DC3000+*avrB* or *avrRpm1* (Grant et al., 2000). We tested aequorin lines of wild type and InsP 5-ptase plants infected with *Pst*DC3000+*avrRpm1.* We found that the early transient rise in [Ca^2+^]_in_ was similar in both wild type and InsP 5-ptase plants but there were differences in the second sustained increase in [Ca^2+^]_in_. The wild type showed a peak of Ca^2+^ at 110 min post inoculation which is similar to the previous report (Grant et al., 2000). In InsP 5-ptase plants, there was a ~20 min delay in the timing of the Ca^2+^ release (Figure 3B) suggesting that InsP_3_-mediated signaling contributes to the cytosolic Ca^2+^ increase observed in avirulent pathogen infected leaves.

### ROS production and SA-mediated signaling during ETI

The oxidative burst in avirulent pathogen infected leaves is part of the disease resistance mechanism (see review in Lamb and Dixon, 1997). It leads to the generation of ROS, which presumably could confine bacterial growth (see review in Torres et al., 2006). To examine the ROS production in avirulent pathogen infected leaves, we used 3,3′-diaminobenzidine (DAB) to detect ROS production *in situ*. Similar to the result observed with flagellin treatment (Figure 1A), no difference in ROS production between wild type and InsP 5-ptase plants was observed (Supplementary Figure 1). In both lines staining was visible at 6 h and disappeared at 48 h either after inoculation or spraying with *Pst*DC3000+*avrRpt2*. The results demonstrate that the HR-induced oxidative burst and ROS production are unaffected in InsP 5-ptase plants and suggest that ROS generation in plant ETI does not involve InsP_3_-mediated pathways.

**Figure 4:**
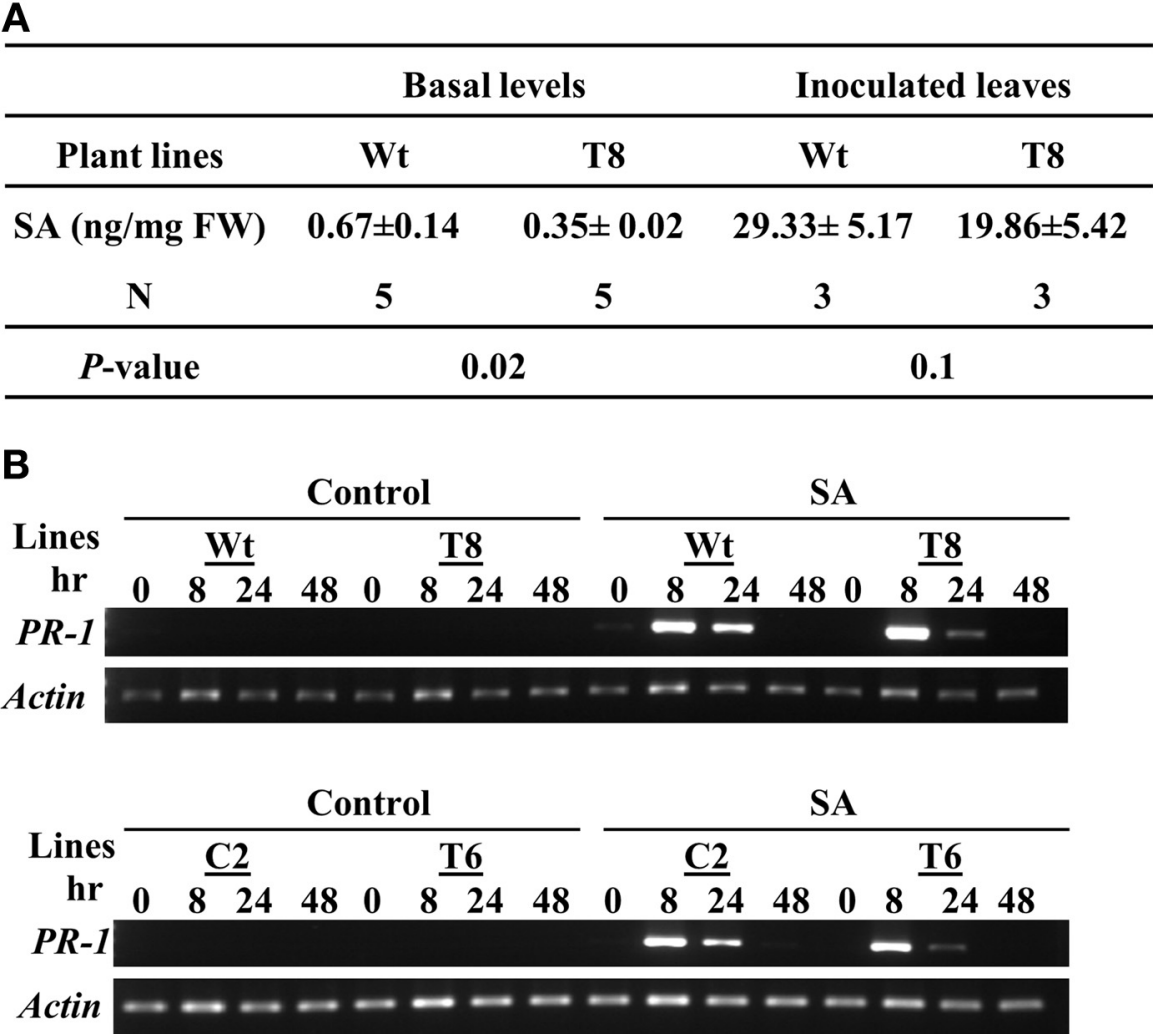
**InsP 5-ptase plants have a low basal level of SA; however the local response to exogenous SA is normal. (A)** SA levels were measured in untreated leaves (basal) or in leaves harvested from plants inoculated with *Pst* DC3000+*avrRpt2* at a concentration of OD_600_ = 0.1 (1 × 10^8^ cfu/ml) at day 2 post-inoculation. Data is the average of three to five experiments ± SE. Each experiment consisted of a pool of leaves from four individual plants/line. **(B)** Wild type (Wt), two independent InsP 5-ptase lines (T6 and T8), and vector control (C2) plants were sprayed with 300 μM of SA or control solution (0.05% ethanol). Leaves were harvested before (0) and at 3, 8, 24, and 48 h after treatment. RT-PCR was carried out with gene-specific primers for *PR-1* and *Actin*.

Because SA is a key systemic signal generated during ETI, we compared the SA levels in wild type and InsP 5-ptase plants (Figure 4A). We measured total SA including the conjugated form. The basal SA level in transgenic plants was ~50% of the amount in wild type. After inoculating with *Pst*DC3000+*avrRpt2*, the SA levels increased in local inoculated wild type leaves and to a lesser extent in the InsP 5-ptase leaves. Although the SA reduction post inoculation in the transgenic plants is not highly significant (*P* = 0.1), the results do show that the total basal SA in InsP 5-ptase plants was significantly lower compared to wild type. It was not impaired as much as in *nahG* plants and *sid2* mutants, but this low efficiency might be enough to reduce the SA-mediated signaling and cause delay in downstream gene expression, such as *PR-1*.

To further investigate whether the delayed *PR-1* gene induction observed in InsP 5-ptase plants (Figure 2D) was due to a delayed response to SA, we applied exogenous SA and monitored *PR-1* gene induction over a two day period. The wild type and vector control plants (C2) as well as two independent InsP 5-ptase lines were treated with 300 μM SA by mist spray, treated leaves were harvested at 0, 8, 24, and 48 h and *PR-1* transcript levels was monitored by RT-PCR (Figure 4B). Both InsP 5-ptase lines have similar induction of *PR-1* as wild type and C2 in response to exogenous SA, suggesting that the InsP 5-ptase plants can respond to SA similar to the wild type. The data suggest that the delay in *PR-1* gene induction is probably due to the delayed synthesis of SA. This result excludes the possibility of InsP_3_ being an intermediate between SA and *PR-1* gene expression.

### The SAR response in InsP 5-ptase plants

**Figure 5:**
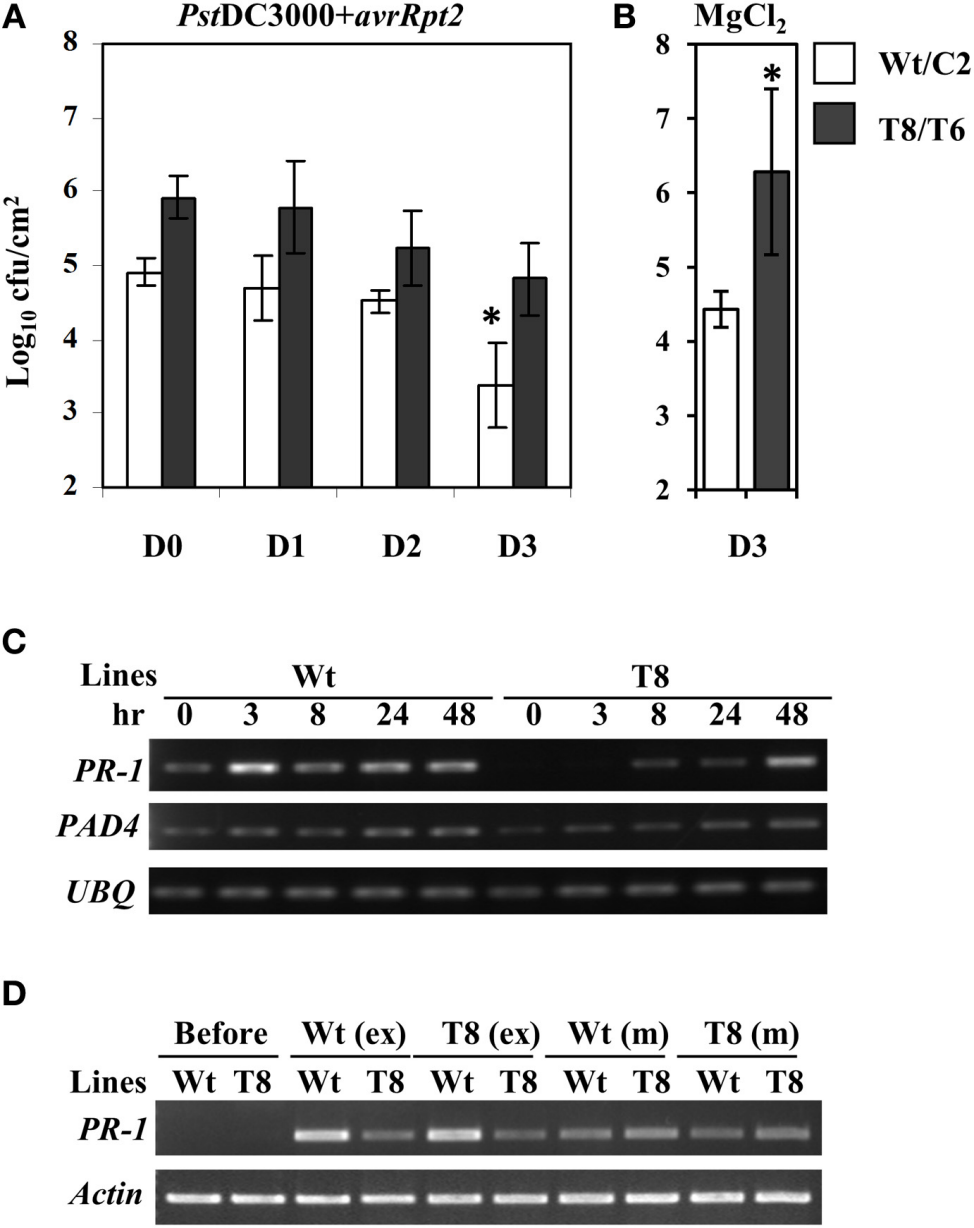
**The SAR response is delayed in the InsP 5-ptase plants**. The SAR assay was carried out using wild type (Wt), vector control (C2) and two independent InsP 5-ptase lines (T6 and T8). Plants were either first inoculated with *Pst* DC3000+*avrRpt2* (OD_600_ = 0.001, 7 × 10^5^ cfu/ml) **(A)**, or 10 mM MgCl_2_ mock solution **(B)**, then sprayed with *Pst*DC3000 (OD_600_ = 0.004, 2 × 10^6^ cfu/ml) at day 0, 1, 2, or 3. Systemic leaves were harvested and the bacterial growth was quantified at day 4 post spray. Data is the average of three experiments ± SD. Each experiment has three plants per line. Results from Wt and C2 (Wt/C2), and T6 and T8 (T8/T6) were pooled for analysis. ^*^P < 0.05 **(C)** Systemic leaves were also harvested before (0) and after initial inoculation at 3, 8, 24, and 48 h for RT-PCR carried out with gene-specific primers for *PR-1*, *PAD4*, and *UBQ10*. **(D)** For the exudate experiment, only Wt and T8 and their reciprocal treatments are shown. Leaves were harvested before or at 48 h post exudate (ex) or mock (m) infiltration for RT-PCR carried out with gene-specific primers for *PR-1* and *Actin*.

In order to examine whether the reduced SA basal levels and delayed *PR-1* induction would compromise SAR in InsP 5-ptase plants, we carried out an SAR assay (Zhang et al., 2010). Plants were first inoculated with *Pst*DC3000+*avrRpt2*, (two leaves per plant), and then challenged with *Pst*DC3000 by spraying whole plants at day 0, 1, 2, and 3 post first inoculation. To monitor the progression of bacterial growth in systemic leaves, bacterial counts were measured in leaves at day 4 post spray. We found that wild type plants acquired resistance to *Pst*DC3000 around day 3 post first inoculation showing a reduced bacterial growth compared to that at day 0, 1, and 2 (Figure 5A). Although the bacterial growth in InsP 5-ptase plants also declined slightly at day 3, the reduced growth is not statistically significant compared to that at day 0, 1, and 2. The assay result indicates that the SAR response was affected in InsP 5-ptase plants. It could be that the acquired resistance was not induced as much as in the wild type at day 3, (i.e., a delayed response), or it could be that the level of resistance was substantially reduced. No acquired resistance was detected when plants were first mock inoculated with 10 mM MgCl_2_ (Figure 5B).

To investigate the expression levels of defense-related genes during SAR induction in systemic leaves, we performed qRT-PCR analysis over a time course. We first inoculated plants with *Pst*DC3000+*avrRpt2* (two leaves per plant), then the systemic leaves were harvested at 3 h, 8 h, day 1, and day 2 post-inoculation. We monitored expression of a total of 47 genes (for a complete gene list see Supplementary Table 1). We found 14 genes that were differentially expressed in the wild type and two transgenic plant lines that were either only induced in the wild type or showed reduced and delayed induction in the transgenic plants (Table 2, calculated data are in Supplementary Table 3). Among these 14 genes, eight of them had low basal expression in the transgenic plants. The remaining six genes, which were not basally low, exhibited a reduced and delayed induction. These genes included *NHL10*, *RLK1*, *PAD4*, and *NIMIN-2*, which are implicated in SAR and SA-mediated signaling (Zhou et al., 1998; Li et al., 2004; Navarro et al., 2004; Weigel et al., 2005). To further confirm the qRT-PCR result, we also performed an independent RT-PCR analysis on systemic leaves (Figure 5C) using the marker genes *PR-1* and *PAD4.* The RT-PCR results were consistent with the qRT-PCR data. The results indicate that the expression of a subset of genes involved in SAR were altered, both in timing and extent in the InsP 5-ptase plants.

**Table 2:** **Genes which were either not induced or showed delayed expression in distal leaves of InsP 5-ptase plants compared to Wt^a^**.

**TAIR ID**	**Gene**
At1g02920	Glutathione S-transferase, putative, ***GST11*^b^**
At1g33960	avrRpt2 induced gene 1, ***AIG1***
At2g02930	Glutathione S-transferase, putative, ***GST16***
At2g35980	NDR1/HIN1 like protein, *NHL10*
At3g26820	Esterase/lipase/thioesterase family protein
At3g50770	Calmodulin-related protein, putative, *CML41*
At3g57260	Glycosyl hydrolase family 17 protein, similar to glucan endo-1,3-beta glucosidase, ***PR-2***
At4g23150	Cysteine-rich receptor-like protein kinase (***RLK7, CRK7***)
At1g75040	Pathogenesis-related protein 5, ***PR-5***
At2g14610	Pathogenesis-related protein 1, ***PR-1***
At2g32680	Receptor like protein 23, ***RLP23***
At3g25882	NPR1/NIM1-interacting protein 2, *NIMIN-2*
At3g52430	Phytoalexin-deficient 4 protein, *PAD4*
At5g60900	Receptor-like protein kinase 1, *RLK1*

a Expression was monitored in distal following inoculation of local leaves with PstDC3000+avrRpt2.

b The names in bold are also basally low and listed in Table 1.

### Signal molecules generated in InsP 5-ptase plants

The onset of SAR requires an array of mobile signals which are generated from the primary infected leaves (reviewed in Gao et al., 2014). These molecules are presumed to be translocated from local inoculated leaves to distal leaves through the phloem tissue. Phloem exudate collected from local leaves provides a means to study the effects of the mobile signals. We performed exudate experiments to examine whether the signal molecules generated from the local infected leaves in wild type can trigger the same response in InsP 5-ptase plants and vice versa. The experimental design is to mimic the process of delivering signal molecules from primary infected leaves to systemic leaves by bypassing the plant delivery system. The exudate from infected plants containing putative signal molecules was collected and injected into healthy uninfected plants. In these experiments, *PR-1* gene expression is expected to increase in the new leaves (similar to systemic leaves) upon injection in response to the presence of the signal molecules. We found that exudates collected from InsP 5-ptase plants were equally effective in inducing *PR-1* gene expression in wild type plants as exudates from wild type plants (Figure 5D). However, InsP 5-ptase plants showed diminished *PR-1* gene expression in response to exudates from either wild type or transgenic plants. These results indicate that the generation of mobile signals at the primary infected site is not impaired in the InsP 5-ptase plants and the exudates were as effective as those produced in wild type. Since the response to exogenous application of SA was also normal in the InsP 5-ptase plants (Figure 4B), these results suggest that InsP 5-ptase plants are affected in either sensing the mobile signal and/or in the synthesis of SA at the systemic site.

## Discussion

**Figure 6:**
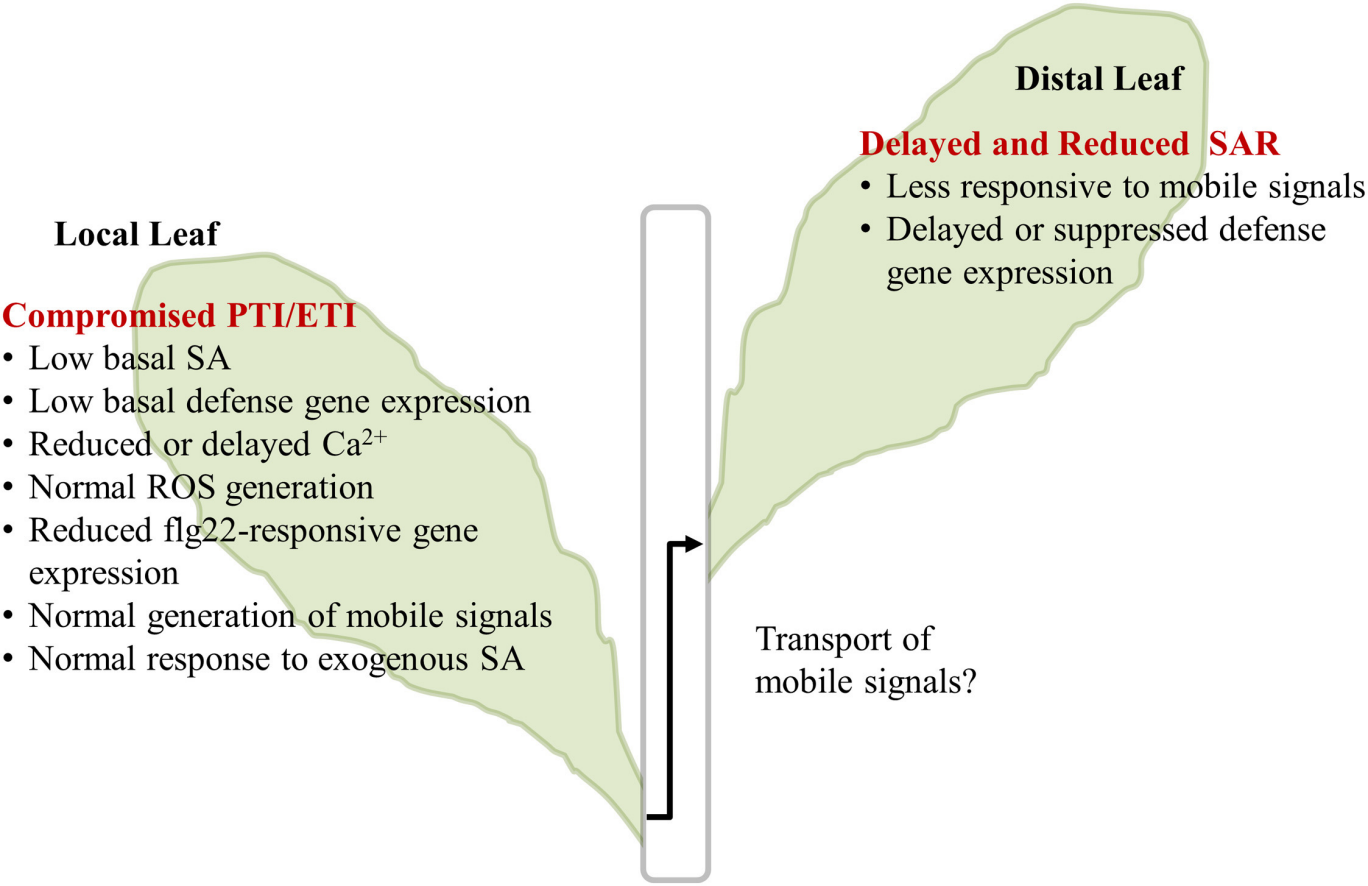
**Summary of InsP 5-ptase plants responses to flg22 and *Pseudomonas syringae***. The major results described in the paper are listed. The transport of mobile signals from local to distal leaves was not investigated and is indicated by the “?.” PTI, PAMP triggered immunity; ETI, Effector triggered immunity; SA, salicylic acid; ROS, reactive oxygen species; SAR, systemic acquired resistance.

A summary of our major results is illustrated in Figure 6. In the InsP 5-ptase plants, PTI responses including MAMP-triggered Ca^2+^ increase and gene expression were affected, although ROS generation was normal. Additionally, ETI responses occurring at the local infected leaves were compromised in InsP 5-ptase plants leading to a reduced and delayed induction of the systemic response.

A reduction in basal InsP_3_ levels is the most dramatic and direct consequence of the constitutive expression of InsP 5-ptase in the InsP 5-ptase transgenic Arabidopsis plants (Perera et al., 2006, 2008). It is reasonable to speculate therefore, that the inability to propagate an InsP_3_ signal is the primary basis for the altered defense responses exhibited by these plants. However, we cannot rule out potential up and downstream effects of the increased turnover of InsP_3_, since InsP_3_ is an intermediate in the phosphoinositide pathway and is linked to both the phospholipids and the inositol phosphates. In previous work, we showed that PIP5K activity and PtdInsP_2_ synthesis was upregulated in transgenic tobacco cells in suspension culture expressing InsP 5-ptase (Perera et al., 2002). Under normal growth conditions, in our hands, we have not detected a up regulation of PtdInsP_2_ synthesis in transgenic Arabidopsis seedlings or plants. It is possible that the InsP 5-ptase plants have altered localized perturbations in membrane phospholipids under specific conditions (such as hydroponic growth, König et al., 2007) or in response to stimuli such as pathogens.

InsP_3_ signaling in plants remains a controversial topic primarily due to the fact that at a molecular level, an InsP_3_ responsive Ca^2+^ channel (analogous to the animal InsP_3_ receptor) has not been identified in plant genomes (discussed in Krinke et al., 2007a; Munnik and Nielsen, 2011). Nevertheless, many studies have demonstrated that InsP_3_ is a physiological ligand (reviewed in Krinke et al., 2007a; Dodd et al., 2010; Pokotylo et al., 2014) and InsP_3_ changes occur rapidly and transiently in response to wide variety of both abiotic and biotic signals/stimuli (reviewed in Krinke et al., 2007a; Im et al., 2010). It has also been suggested that InsP_6_ rather than InsP_3_ is the ligand responsible for Ca^2+^ release from intracellular plant stores (Lemtiri-Chlieh et al., 2003). Since InsP_3_ is an intermediate in the lipid-dependent route of InsP_6_ biosynthesis (reviewed in Gillaspy, 2011, 2013), increased InsP_3_ turnover could be expected to affect InsP_6_ levels and indeed the InsP 5-ptase plants have lower InsP_6_ levels compared to wild type (Perera et al., 2008). Increased susceptibility to several microbial pathogens was reported in low InsP_6_ mutants (Murphy et al., 2008). However, (unlike the InsP 5-ptase plants), these mutants had normal basal levels of SA and normal SA induction upon infection. Therefore we cannot attribute all of the attenuated defense responses of the InsP 5-ptase plants to a decrease in InsP_6_. Other inositol phosphate intermediates, including InsP_4_ and InsP_5_ are implicated in stress responses (reviewed in Pokotylo et al., 2014). It is conceivable that InsP_6_ (and/or other inositol phosphates) act synergistically with InsP_3_ to mediate signaling leading to defense responses.

### Attenuation of Ca^2+^ signals

It is clear that both PTI and ETI share similar signal transduction components and downstream targets (discussed in Ma and Berkowitz, 2007; Tsuda and Katagiri, 2010) and may represent an overlapping and interconnected network. The complexity of this signaling network is illustrated by the convergence and crosstalk between parallel branches (Boudsocq and Sheen, 2013). A rise in cytosolic Ca^2+^ is one common signaling element in response to both PTI and ETI and Ca^2+^ may be a primary signal essential for initiating many of the early events; however there are differences in the timing and duration of the PTI and ETI associated Ca^2+^ signals. Additionally there are feedback loops that modulate the propagation of the Ca^2+^ signal (Ma and Berkowitz, 2007, 2011).

We showed that the InsP 5-ptase seedlings have a greatly attenuated Ca^2+^ signal in response to flg22 compared to wild type (Figure 1B). A similar result was reported using intact leaves exposed to flg22 (Ma et al., 2012). Intriguingly, the InsP 5-ptase plants exhibited a normal Ca^2+^ response to the plant derived peptide elicitor Pep3 (Ma et al., 2012). The Pep receptor PEPR is linked to the cyclic nucleotide gated cation channel CNGC2 (Qi et al., 2010; Ma et al., 2012). Interestingly, the *cngc2* mutant showed an opposite Ca^2+^ response to the InsP 5-ptase plants; an attenuated Ca^2+^ rise in response to Pep3 but a normal Ca^2+^ response to flg22 (Ma et al., 2012). This result supports the involvement of different pathways of Ca^2+^ influx in response to Pep and flg22. It was suggested that Pep primarily targets extracellular stores (via influx by CNGC2) while flg22 may involve an additional contribution from intracellular stores. Detailed studies of the amplitude and kinetics of flg22 dose-dependent Ca^2+^ changes also suggested that two processes or two different stores may be involved in the flg22 induced Ca^2+^ response (Jeworutzki et al., 2010; Ranf et al., 2011).

A biphasic Ca^2+^ response was previously reported in response to avirulent pathogens (Grant et al., 2000). Ma and Berkowitz (2007) proposed that the initial pathogen-associated Ca^2+^ rise is primarily via influx from the apoplast while the subsequent Ca^2+^ rise is from intracellular stores. We observed that while the initial Ca^2+^ peak was similar to wild type, the timing of the second Ca^2+^ peak was delayed in the InsP 5-ptase plants (Figure 3B) which supports a role for InsP_3_ in sustaining and propagating the pathogen-associated cytosolic Ca^2+^ signal.

Our results are consistent with a model in which InsP_3_ (and or its derivatives) may act in an intracellular Ca^2+^ relay which would be downstream of the initial Ca^2+^ influx from the apoplast. Because MAMP-triggered ROS was unaffected in the InsP 5-ptase plants we propose that the initial rise in cytosolic Ca^2+^ in response to MAMP elicitation may activate some downstream targets (such as CDPKs) as well as trigger the early oxidative burst. Alternatively, the ROS response may be “primed” by BIK1 in a parallel Ca^2+^ independent manner (Kadota et al., 2014; Li et al., 2014).

In plants several intracellular compartments serve as Ca^2+^ stores (reviewed in Dodd et al., 2010). The InsP_3_ responsive Ca^2+^ store is likely to be the ER or vacuole; however we cannot rule out the involvement of the chloroplast (Manzoor et al., 2012). A plant specific Ca^2+^ sensor protein CAS, which is located in the chloroplasts, was recently shown to play a role in both PTI and ETI (Nomura et al., 2012). There are shared similarities in the response of the *cas-1* mutant and the InsP 5-ptase transgenic plants in defense signaling, such as increased susceptibility to both virulent and avirulent bacterial strains, basally reduced *PR-1* gene expression and normal ROS generation in response to flg22. However at present, we have no information on whether InsP_3_ (and/or InsP_6_) may affect Ca^2+^ stores in the chloroplast. The basal down regulation of *PR* genes and the reduced activation of *PR* gene expression in the InsP 5-ptase plants may also reflect alterations in ER Ca^2+^ homeostasis as discussed further below.

### SA biosynthesis and SA mediated signaling

InsP 5-ptase plants have low basal SA (under normal control conditions) and when infected with avirulent pathogens, SA levels were lower than wild type plants at day 2 post inoculation (Figure 4), suggesting that SA biosynthesis maybe reduced in the transgenic plants. The reduced levels of SA in InsP 5-ptase plants suggest a link between PI-metabolism and SA accumulation/pathway through a yet unknown mechanism that ultimately affects plant defense, either directly or indirectly. At a transcriptional level, based on previous microarray results (Perera et al., 2008), we have not detected lower expression of the two key genes involved in SA biosynthesis, namely phenylalanine ammonia lyase (*PAL*) and isochorismate synthase (*ICS1*), (Wildermuth et al., 2001). ABA has been implicated as a negative regulator of SA due to their overlapping biosynthetic pathways (de Torres Zabala et al., 2009; reviewed in Cao et al., 2011). However, we did not detect increased basal ABA levels in InsP 5-ptase plants (Perera et al., 2008). Another possibility is that expression of genes encoding positive regulators of SA accumulation (reviewed in Lu, 2009; Ng et al., 2011), could be impaired in InsP 5-ptase plants, resulting in lowered SA levels. One of these genes is *PAD4*, which showed a delayed induction in systemic leaves (Table 2). Similar to InsP 5-ptase plants, mutants of this group of genes have reduced SA accumulation and enhanced susceptibility to pathogens which could be rescued by exogenous SA treatment (Zhou et al., 1998). Recent studies also point to the involvement of Ca^2+^/calmodulin (CaM) in modulating SA biosynthesis. SARD1 and CBP60g, are two related transcription factors which control SA biosynthesis by regulating *ICS1* expression. Interestingly, CBP60g is a calmodulin-binding protein and its activity is Ca^2+^-dependent (Zhang et al., 2010; Truman and Glazebrook, 2012). It is possible that the reduced Ca^2+^ response in InsP 5-ptase plants might affect induction and accumulation of SA via CBP60g activity.

### Attenuated gene expression

We have reported that select early flg22 responsive genes showed reduced expression at 120 min in the InsP 5-ptase plants (Figure 1E). We suspect that some downstream transcriptional responses may require the continued propagation of the Ca^2+^ signal.

We have also shown that the InsP 5-ptase plants have basally reduced levels of some defense-related genes (Table 1). Additionally, induction of *PR-1* was delayed in local leaves and several SAR related genes were either not induced or delayed in induction in systemic leaves (Figure 2D, Table 2). We suggest that these genes are in part under the control of SA and Ca^2+^/CaM which can regulate transcription by either directly or indirectly interacting with transcription factors (review Dong, 2004; Kim et al., 2009; Reddy et al., 2011). The DNA-binding activity of the transcription factor CAMTA (or SR) is enhanced by Ca^2+^/CaM binding (review, Finkler et al., 2007). Arabidopsis CAMTA3 acts as a negative regulator of biotic stress responses. The loss-of-function mutants have elevated levels of SA and exhibit enhanced disease resistance. Additionally, several defense-associated genes (including PR genes) are found to be up-regulated in *camta3* mutants (Galon et al., 2008; Du et al., 2009).

The cyclic nucleotide-gated channel (CNGC) mutant *cngc2* also shows constitutively high *PR-1* gene expression and high SA production (Chan et al., 2008). The phenotypes of both the *camta3* and *cngc2* mutants are opposite to what we observed in the InsP 5-ptase plants, which have low *PR-1* gene expression and low SA levels. We have noted a striking inverse correlation between genes that are basally down regulated in the InsP 5-ptase plants and genes that are upregulated in *camta3* and *cngc2* (Supplementary Table 4). Twelve of the basally upregulated genes in *cngc2*, and thirteen of the upregulated genes in *camta3* were found downregulated in InsP 5-ptase plants (Perera et al., 2008) including *PR-1*, *PR-2*, and *PR-5* (Supplementary Table 4). Furthermore, there was strong overlap between this subset of genes and the top 50 candidates in the *PR-1* coexpression network as well as with genes that upregulated in wild type seedlings grown under high Ca^2+^ conditions (Chan et al., 2008). These results are further support for Ca^2+^ mediated regulation of gene expression although at present, the exact mechanism of activation of these over lapping genes is not known.

SA is also implicated in the regulation of *PR* gene expression via the activation of the TGA (TGACG Motif-Binding Factor) transcription factors (review, Dong, 2004; Fu and Dong, 2013). Accumulation of SA affects the cellular redox and controls the translocation of the cofactor NPR1 into the nucleus where it binds and activates TGAs. Most TGAs act as positive regulators to induce PR genes as well as the expression of genes encoding ER resident proteins involved in protein folding and Ca^2+^ storage. The induction of the protein secretory pathway is required for SAR (Wang et al., 2005). InsP 5-ptase plants have low basal levels of *PR-1*, *PR*-5, *CRT3*, and *BiP3* (Table 1), which may be in part due to low basal SA and/or low ER Ca^2+^.

In conclusion, we show that the constitutive expression of InsP 5-ptase affects Ca^2+^ release, expression of a subset of defense related genes and both basal and SAR responses but does not affect ROS production. We favor the model postulated by Ranf et al. (2011) in which Ca^2+^ acts as an “on/off switch” and a threshold level of Ca^2+^ is required for full activation of the downstream pathways. The InsP 5-ptase plants are impaired in their ability to propagate and maintain the Ca^2+^ signal and therefore are unable to mount a full and robust defense response.

## Author contributions

Imara Y. Perera and Chiu-Yueh Hung designed and supervised the experiments and constructed the final manuscript. Chiu-Yueh Hung, Peter Aspesi Jr, and Aaron W. Lomax carried out the pathogen infection and SAR experiments; Chiu-Yueh Hung and Melissa R. Hunter designed and carried out qRT-PCR and analysis. Aaron W. Lomax also prepared samples for SA analysis. Imara Y. Perera supervised and carried out the flg22 treatment, ROS, and Ca^2+^ assays.

### Conflict of interest statement

The authors declare that the research was conducted in the absence of any commercial or financial relationships that could be construed as a potential conflict of interest.
