# Supplementary material for: Phosphoinositide-signaling is one component of a robust plant defense response
Source: Front Plant Sci. 2014 Jun 11;5:267. doi: 10.3389/fpls.2014.00267 (PMC4052902; doi:10.3389/fpls.2014.00267)
Supplement: Supplementary file 1 [file Presentation1.ZIP › Supp Tables 1-4.docx]

**SUPPLEMENTRAY TABLES**

**Supplementary Table 1 │ List of selected genes from Sigma Primer Library for Arabidopsis Pathogen-inducible Genes (PN# PR0100)**

| **Categories** | **Well#** | **TAIR ID** | **Protein encoded** |
| --- | --- | --- | --- |
| **GST** | F1 | At1g02920 | glutathione S-transferase (GST11) |
|  | F7 | At2g02930 | glutathione S-transferase (GST16) |
|  | D8 | At1g28480 | a member of the glutaredoxin (GRX480) |
|  | D6 | At1g65970 | thioredoxin-dependent peroxidase 2 (TPX2) |
|  |  |  |  |
| **ethylene** | D2 | At4g17490 | ERF subfamily B-3 of ERF/AP2 transcription factor family (ERF6) |
|  | G10 | At4g11280 | 1-aminocyclopropane-1-carboxylate (ACC) synthase (ACS6) |
|  | C12 | At4g17500 | ERF subfamily B-3 of ERF/AP2 transcription factor family (ATERF-1) |
|  | C6 | At3g04640 | glycine-rich protein, response to ethylene and wounding |
|  |  |  |  |
|  |  |  |  |
| **lipase** | D4 | At3g26820 | esterase/lipase/thioesterase family protein |
|  | A9 | At4g11830 | phospholipase D gamma 2 / PLD gamma 2 (PLDGAMMA2). |
|  | H2 | At2g39400 | hydrolase, alpha/beta fold family protein |
|  |  |  |  |
| **phosphorylation** | D7 | At4g24340 | phosphorylase family protein |
|  | C4 | At1g47510 | phosphatidylinositol polyphosphate 5-phosphatase (AT5PTASE11) |
|  |  |  |  |
| **Ca/CaM binding** | A5 | At1g18890 | calcium-dependent protein kinase 1 (CDPK1) |
|  | D3 | At3g50770 | calmodulin-like 41 (CML41) |
|  | D9 | At1g73805 | encodes SAR Deficient 1 (SARD1), a calmodulin-binding protein |
|  | C11 | At5g26920 | calmodulin-binding protein (CBP60g) |
|  |  |  |  |
| **zinc finger** | F10 | At2g40140 | zinc finger (CCCH-type) family protein (SZF2) |
|  | G3 | At4g11370 | zinc finger (C3HC4-type RING finger) family protein (RHA1a) |
|  | G7 | At5g59820 | zinc finger (C2H2 type) family protein (ZAT12) |
|  | G11 | At3g28210 | zinc finger protein (PMZ, SAP12) |
|  | E8 | At3g46090 | zinc finger (C2H2 type) family protein (ZAT7) |
|  |  |  |  |
| **WRKY** | C1 | At3g56400 | WRKY family transcription factor (WRKY70) |
|  | G9 | At2g38470 | WRKY family transcription factor (WRKY33) |
|  |  |  |  |
| **MYB** | C2 | AT2g31180 | myb family transcription factor (MYB14) |
|  | G12 | At4g37260 | myb family transcription factor (MYB73) |
|  |  |  |  |
| **Protein Kinase** | B11 | At4g36950 | mitogen-activated protein kinase kinasekinase 21 (MAPKKK21) |
|  | D5 | At4g23150 | cysteine-rich receptor-like protein kinase (CRK7, RLK7) |
|  | E4 | At4g11890 | receptor-like cytosolic kinase ARCK1, CRK45 |
|  | F12 | At4g26070 | mitogen-activated protein kinase kinase (MAPKK) (MKK1) (MEK1) |
|  | H8 | At1g05100 | mitogen-activated protein kinase kinasekinase 18 (MAPKKK18) |
|  | A1 | At2g32680 | receptor like protein 23 (RLP23), has kinase activity |
|  | B4 | At2g31880 | leucine-rich repeat transmembrane protein kinase, EVERSHED (EVR) |
|  | G1 | At2g13790 | somatic embryogenesis receptor-like kinase 4 (SERK4) |
|  | A6=E7 | At3g16530 | Lectin like protein kinase |
|  | A4=B7 | At5g60900 | G-type lectin S-receptor-like serine/threonine-protein kinase (RLK1) |
|  | B5 | At3g59700 | L-type lectin-domain containing receptor kinase (LECRK1) |
|  | B8 | At4g23180 | cysteine-rich receptor-like protein kinase 10 (CRK10) (RLK4) |
|  | E5 | At4g23130 | cysteine-rich receptor-like protein kinase (CRK5) (RLK6) |
|  |  |  |  |
| **Defense gene** | E1 | At3g57260 | beta 1,3-glucanase (PR-2) |
|  | E3 | At2g14610 | pathogenesis-related protein 1 (PR-1) |
|  | H1 | At3g52430 | phytoalexin-deficient 4 protein (PAD4) |
|  | H6 | At3g25882 | NPR1/NIM1-interacting protein 2 (NIMIN-2) |
|  | A7 | At5g44420 | plant defensin protein, putative (PDF1.2a) |
|  | B1 | At3g04720 | hevein-like protein (HEL)(PR-4) |
|  |  |  |  |
| **Others** | H9 | At1g66700 | Arabidopsis SABATH methyltransferase gene family (PXMT1) |
|  | D1 | At5g47200 | Rab GTPase (AtRabD2b ) |

**Supplementary Table 1 (Cont)**

**Supplementary Table 2│ Primer sequence information**

| TAIR ID | Gene | F/R | Sequence 5’-3’ |
| --- | --- | --- | --- |
| At1g75040 | *PR-5* | F  R | CGGCGATGGAGGATTTGAATTGAC  CGCCGCCGTTACATCTTAGACC |
| At1g33960 | *AIG1* | F  R | CGCAGCATTGATGAAGGAGTTACAG  TGAGCATTTGCGCTTCCATTTCAG |
| At1g64280 | *NPR1* | F  R | CGCACTCATGATCGCAAAACAAG  TGCTTCCGTTGGAAAAAGACGTT |
| At2g35980 | *NHL10* | F  R | TTCCTGTCCGTAACCCAAAC  CCCTCGTAGTAGGCATGAGC |
| At1g61560 | *MLO6* | F  R | CTGGCTACCGTTCATTCCCTTCATC  TAGCGGTGTGCCTTTCACTACGTC |
| At1g02930 | *GST1* | F  R | TCGAGCTCAAAG GTGGTGAACACA  TGGGTCAAACTCATGCGACTCA |
| At1g51850 | *RLK* | F  R | GAGCATCACCCAAGCCATTCAGA  TGAGGGAGGAACTGAGCCACTTAGA |
| At2g19190 | *FRK1* | F  R | ATCTTCGCTTGGAGCTTCTC  TGCAGCGCAAGGACTAGAG |

**Supplementary Table 3│ QRT-PCR of systemic leaves** ^a^**.**

| **TAIR ID** | **Protein Encoded** | **Wt** | | | | **T8** | | | |
| --- | --- | --- | --- | --- | --- | --- | --- | --- | --- |
|  |  | **0** | **3h** | **24h** | **48h** | **0** | **3h** | **24h** | **48h** |
| At1g02920 | GST11 | 1 | **2.8** | 0.8 | 1.3 | 0.3 | 0.5 | 0.4 | **1.7** |
| At2g02930 | GST16 | 1 | **2.2** | 0.8 | 0.6 | 0.5 | 0.6 | 1.0 | 1.0 |
| At2g35980 | NHL10 | 1 | **4.6** | 0.7 | 0.5 | 0.4 | 0.7 | 0.5 | 0.3 |
| At3g26820 | esterase/lipase  /thioesterase | 1 | **8.6** | 0.9 | 1.1 | 0.5 | 1.1 | 0.5 | 1.0 |
| At3g50770 | CML41 | 1 | **2.1** | 0.5 | 0.5 | 0.5 | 1.0 | 0.4 | 0.5 |
| At4g23150 | RLK7 | 1 | **2.0** | 1.1 | 1.1 | 0.1 | 0.2 | 0.3 | 0.6 |
| At3g52430 | PAD4 | 1 | **1.6** | 1.3 | 0.9 | 0.5 | 0.4 | 1.0 | 1.3 |
| At5g60900 | RLK1 | 1 | **1.9** | 1.7 | 1.1 | 0.9 | 0.5 | **1.6** | **1.6** |
| At1g33960 | AIG1 | 1 | **5.4** | **1.9** | **4.1** | 0.2 | 0.8 | 0.4 | 0.8 |
| At2g14610 | PR-1 | 1 | **7.5** | **3.2** | **2.9** | 0.02 | 0.07 | 0.3 | **1.3** |
| At3g57260 | PR-2 | 1 | **6.6** | **2.8** | **1.7** | 0.1 | 0.6 | 0.3 | **1.5** |
| At1g75040 | PR-5 | 1 | **7.4** | **3.5** | **5.5** | 0.2 | 0.7 | 0.8 | **3.3** |
| At2g32680 | RLP23 | 1 | **2.3** | **2.3** | 1.0 | 0.1 | 0.1 | 0.6 | **1.8** |
| At3g25882 | MIMIN-2 | 1 | **3.1** | **4.1** | **4.1** | 0.4 | 0.8 | **5.1** | **5.8** |

^a^ Data is the average fold change compared to wild type 0 time control from five independent experiments. Numbers in bold indicate gene expression is induced. Similar trends were observed with transgenic line T6.

**Supplementary Table 4│ Comparison showing overlap between genes that are basally down in InsP 5-ptase plants and up-regulated in other mutants or experimental conditions**

| **Protein Description** | **InsP 5-ptase^a^**  **down** | ***PR-1*^b^ coexpressed**  **Top 50** | ***cngc2*^c^**  **up** | **WS+Ca^2+d^**  **up** | ***camta3*^e^**  **up** |
| --- | --- | --- | --- | --- | --- |
| pathogenesis-related protein 1 (PR-1) | At2g14610 | x | x |  | x |
| expansin family protein (EXPR3) | At2g18660 | x | x | x | x |
| pathogenesis-related protein 5 (PR-5) | At1g75040 | x | x | x | x |
| cysteine-rich receptor-like protein kinase (CRK7, RLK7) | At4g23150 | x | x | x | x |
| chitinase, putative | At2g43570 | x | x |  | x |
| expressed protein | At1g14870 | x | x |  | x |
| beta 1,3-glucanase (PR-2) | At3g57260 | x | x | x | x |
| cysteine-rich receptor-like protein kinase (CRK37) | At4g04500 | x | x |  |  |
| receptor-like protein kinase 5 (RLK5) | At4g23140 | x | x | At4g23130 | x |
| oxidoreductase, 2OG-Fe(II) oxygenase family protein | At5g24530 | x |  | x | x |
| heavy-metal-associated domain-containing protein | At5g52740 |  | x |  |  |
| cytochrome P450 71A13, putative (CYP71A13) | At2g30770 |  | x |  |  |
| protease inhibitor/seed storage/lipid transfer protein (LTP) family protein | At5g55450 | x |  | x | x |
| alpha-amylase, putative / 1,4-alpha-D-glucan glucanohydrolase, putative | At4g25000 |  |  | x |  |
| vacuolar sorting receptor 6 (VSR6) | At1g30900 |  |  |  | x |
| calreticulin 3 (CRT3) | At1g08450 | x |  |  | x |
| cation exchanger, putative (CAX3) | At3g51860 |  |  | x |  |
| aspartyl protease family protein | At5g10760 | x | x | x | x |

^a^: The data is obtained from Perera et al., 2008.

^b^: http://atted.jp (Obayashi et al., 2009).

^c^: The data is obtained from Chan et al., 2008.

^d^: The data is obtained from Chan et al., 2008.

^e^: The data is obtained from Galon et al., 2008
