# Supplementary figures and images for: Phosphoinositide-signaling is one component of a robust plant defense response
Source: Front Plant Sci. 2014 Jun 11;5:267. doi: 10.3389/fpls.2014.00267 (PMC4052902; doi:10.3389/fpls.2014.00267)

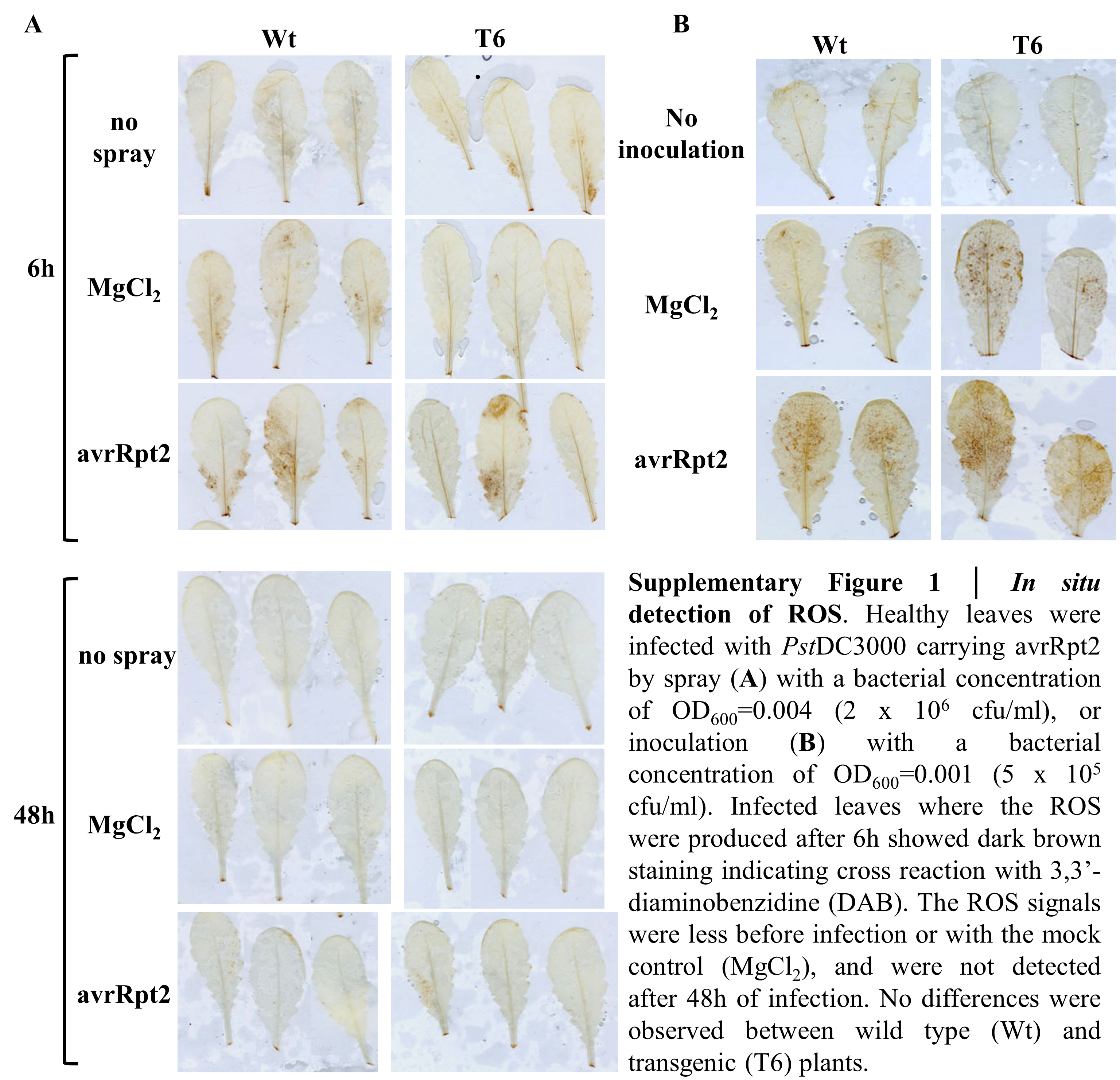

Supplement: Supplementary file 1 [file Presentation1.ZIP › Supp Fig 1.TIF]
